# Immunization Patterns of Children with Chronic Neurological and Neurodevelopmental Disorders: A Cross-Sectional Study from Istanbul

**DOI:** 10.3390/children13070891

**Published:** 2026-07-02

**Authors:** Emek Uyur, Merve İşeri Nepesov, Nilüfer Eldeş Hacıfazlıoğlu, Nihan Uygur Külcü, Rabia Gönül Sezer Yamanel

**Affiliations:** 1 Department of Pediatric Neurology, Zeynep Kamil Maternity and Children’s Diseases Training and Research Hospital, University of Health Sciences, 34668 Istanbul, Türkiye; nilufereldes.hacifazlioglu@sbu.edu.tr; 2 Department of Pediatric Infectious Diseases, Zeynep Kamil Maternity and Children’s Diseases Training and Research Hospital, University of Health Sciences, 34668 Istanbul, Türkiye; merve.iserinepesov@saglik.gov.tr; 3 Department of Pediatrics, Zeynep Kamil Maternity and Children’s Diseases Training and Research Hospital, University of Health Sciences, 34668 Istanbul, Türkiye; nihan.uygurkulcu@saglik.gov.tr (N.U.K.); rabiagonul.sezer@sbu.edu.tr (R.G.S.Y.)

**Keywords:** immunization, autism spectrum disorder, cerebral palsy, Down syndrome, neurodevelopmental disorders, pediatric neurology, vaccine hesitancy, vaccination continuity

## Abstract

**Highlights:**

**What are the main findings?**

- Partial vaccination was substantially more common than complete vaccine refusal among children with chronic neurological and neurodevelopmental disorders.
- Vaccination completeness and the age period after which routine immunization was discontinued differed significantly across diagnostic groups.

**What are the implications of the main findings?**

- Incomplete immunization in children with chronic neurological and neurodevelopmental disorders appears to follow diagnosis-specific patterns rather than a uniform trend.
- Assessing vaccination continuity may help support diagnosis-sensitive immunization counseling during pediatric neurology follow-up.

**Abstract:**

**Background/Objectives:** Children with chronic neurological and neurodevelopmental disorders (CNDD) constitute a medically vulnerable population in whom routine childhood immunization may be interrupted because of clinical complexity and parental concerns. This study aimed to evaluate immunization patterns, timing of vaccination interruption, and associated clinical and sociodemographic factors in children with CNDDs followed at a tertiary pediatric neurology center. **Methods:** This prospective cross-sectional study included 545 children aged 1–18 years with chronic neurological and neurodevelopmental disorders evaluated between September and November 2025. Immunization status according to the Turkish National Immunization Program was classified as fully vaccinated, partially vaccinated, or not vaccinated. Information regarding non-NIP vaccines, timing of vaccination interruption, parental characteristics, and motor impairment severity assessed using the Gross Motor Function Classification System (GMFCS) was collected. Associations between vaccination status and clinical variables were evaluated using chi-square tests and logistic regression analysis. **Results:** Overall, 72.1% of children were fully vaccinated, 25.0% were partially vaccinated, and 2.9% had never received routine childhood vaccines. Immunization status differed significantly across diagnostic groups (*p* < 0.001). Partial vaccination was more common than complete vaccine refusal across most diagnostic categories. Lower full vaccination rates were observed in autism spectrum disorder and cerebral palsy, whereas higher coverage was observed in Down syndrome. Among partially vaccinated children, interruption of routine immunization after 48 months was the most frequent pattern. In multivariable analysis, only maternal age remained significantly associated with incomplete or no vaccination. **Conclusions:** Incomplete immunization in children with CNDDs was more commonly characterized by partial vaccination than complete vaccine refusal and followed diagnosis-specific patterns. Evaluating vaccination continuity during pediatric neurology follow-up may help support diagnosis-sensitive immunization counseling.

## 1. Introduction

Routine childhood immunization represents one of the most effective strategies for preventing infectious diseases and reducing childhood morbidity and mortality worldwide. Despite substantial advances in global vaccination programs, gaps in immunization coverage persist. Recent international estimates indicate that approximately 14.3 million children did not receive any routine vaccines during 2023–2024, and that coverage of key indicators, such as the third dose of diphtheria–tetanus–pertussis (DTP3), remains below optimal levels. These gaps continue to pose a risk for the re-emergence of vaccine-preventable diseases, particularly among vulnerable populations [1,2].

Children with chronic neurological and neurodevelopmental disorders (CNDDs) constitute a high-risk population due to increased medical complexity, frequent healthcare utilization, and greater susceptibility to severe infectious outcomes [3]. These disorders comprise a heterogeneous group of conditions influenced by complex genetic, molecular, and environmental mechanisms, contributing to substantial developmental vulnerability and diverse long-term healthcare needs [4]. Although timely vaccination is particularly important in this group, immunization practices may be influenced by parental concerns regarding vaccine safety and fear of neurological deterioration [5]. In addition, physician-recommended postponement of vaccination has been reported in certain clinical contexts, further contributing to delays or incomplete immunization. These factors, together with increasing public debate surrounding vaccine safety, may contribute to delayed, incomplete, or refused vaccination in children with chronic neurological conditions [6].

Available evidence suggests that vaccination practices in children with chronic neurological disorders may be delayed or incomplete in certain subgroups compared with healthy peers. However, existing studies have largely focused on selected diagnostic groups—most commonly cerebral palsy or autism spectrum disorder—and have not comprehensively addressed vaccination timing or caregiver-reported reasons for incomplete immunization across a broader spectrum of neurodevelopmental conditions [6,7,8,9].

In Türkiye, routine childhood vaccinations are provided free of charge through the National Immunization Program (NIP), and overall vaccination coverage in the general population is high [1,10]. Nevertheless, real-world data focusing specifically on children with chronic neurological and neurodevelopmental disorders remains limited. In particular, the combined effects of diagnostic category, motor impairment severity, and sociodemographic characteristics on immunization patterns have not been sufficiently explored.

Therefore, this study aimed to evaluate immunization patterns in a heterogeneous cohort of children with chronic neurological and neurodevelopmental disorders followed at a tertiary pediatric neurology center in Istanbul, with a particular focus on vaccination completeness, timing, and associated clinical and sociodemographic factors.

## 2. Materials and Methods

### 2.1. Study Design and Setting

This prospective cross-sectional observational study was conducted at the Child Neurology Department of Zeynep Kamil Maternity and Children’s Diseases Training and Research Hospital, including both outpatient and inpatient settings.

### 2.2. Participants

Children aged between 1 and 18 years who were followed for chronic neurological or neurodevelopmental disorders at the Child Neurology Department between 1 September 2025 and 1 November 2025 were eligible for inclusion. Participants were enrolled consecutively during the predefined study period, and study data were collected prospectively at the time of routine outpatient visits or inpatient admissions. Patients for whom vaccination status could not be verified through parental reports supported by vaccination cards and/or available medical records, as well as those whose parents or legal guardians declined participation, were excluded from the study. During the planned study period, 613 children met the eligibility criteria for chronic neurological or neurodevelopmental disorders. Of these, 12 were excluded because vaccination status could not be verified, and 56 families declined participation. Therefore, 545 children were included in the final analysis, corresponding to an enrollment rate of 88.9% among eligible patients.

The study population comprised a broad spectrum of chronic neurological and neurodevelopmental conditions, including cerebral palsy, autism spectrum disorder, spina bifida, neuromuscular disorders, hydrocephalus, neurometabolic disorders, Down syndrome, and other neurogenetic disorders. Because of its relatively high prevalence in the study cohort, Down syndrome was analyzed as a separate diagnostic category. Patients with syndromic, monogenic, chromosomal, or neurodevelopmental genetic conditions were collectively classified under neurogenetic disorders.

In cases of multiple diagnoses, patients were classified according to a predefined hierarchical approach based on the primary etiological diagnosis. Accordingly, when a confirmed genetic diagnosis was present, the underlying genetic condition was prioritized over secondary phenotypic manifestations. Down syndrome was therefore prioritized when present, and children with cerebral palsy who had an identified genetic etiology were categorized under neurogenetic disorders.

### 2.3. Data Collection Procedures

Clinical evaluation was performed as part of routine care. Parents or legal guardians who agreed to participate provided written informed consent and completed a brief structured questionnaire, which required approximately five minutes to complete.

The questionnaire collected demographic information, including age, sex, birth characteristics, parental education level, and number of children in the household. Clinical data—comprising the primary neurological diagnosis, duration of illness, level of motor impairment assessed using the Gross Motor Function Classification System (GMFCS), current medical treatments, and history of hospitalizations—were obtained from medical records. Gross motor function was classified using the Gross Motor Function Classification System (GMFCS).

Although originally developed for children with cerebral palsy, the GMFCS was used in this study as a standardized measure of functional motor impairment across diagnostic groups. Given the heterogeneous nature of the study population, no single disease-specific motor classification system could be consistently applied across all included diagnostic groups. The primary purpose of using the GMFCS was not to assess disease-specific severity or to compare disease severity across diagnostic groups, but to categorize overall motor impairment severity for analytical purposes. For analytical purposes, children were categorized as having normal motor development, mild–moderate motor impairment (GMFCS levels I–III), or severe motor impairment (GMFCS levels IV–V).

Information on vaccination status according to the Turkish National Immunization Program was obtained from parental reports, supported by vaccination cards, and, when available, cross-checked against electronic hospital records and the national electronic health system (e-Nabız). Because vaccination records may be updated at different time points across available documentation sources during routine clinical practice, the most complete and up-to-date documented vaccination record available at the time of data collection was used for the classification of vaccination status. Vaccination status and interruption patterns were determined based on these documented records. Therefore, delayed doses that were subsequently administered were captured in the available records and incorporated into the vaccination classification.

### 2.4. Evaluation of Vaccination Status

Routine childhood immunization status was classified as fully vaccinated, partially vaccinated, or never vaccinated in accordance with the Turkish National Immunization Program (TNIP). The national immunization schedule used for classification is provided in Supplementary Table S1. For children who were not fully vaccinated, the reason for incomplete immunization was recorded as either parental concern regarding potential neurological worsening or temporary postponement recommended by a physician.

Information on the administration of non-National Immunization Program (non-NIP) vaccines, including rotavirus, meningococcal, and influenza vaccines, was also collected.

Among children who were not fully vaccinated, vaccination continuity was evaluated based on the age period after which routine childhood immunizations were no longer received, rather than on individual missed vaccine doses. Given the heterogeneity of vaccination patterns within the cohort, incomplete vaccination was defined as discontinuation of routine childhood immunization. Children were categorized into four groups: those who did not receive routine vaccines from 12 months onward, from 24 months onward, or from 48 months onward, and those in whom vaccination continued beyond 48 months but was subsequently interrupted. This classification reflects the age threshold after which scheduled routine vaccinations were no longer administered. These age categories were selected to reflect key milestones within the Turkish National Immunization Program, corresponding to completion of the primary infant vaccination series, early childhood booster vaccinations, and preschool booster vaccinations, respectively (Supplementary Table S1).

### 2.5. Statistical Analysis

All analyses were conducted using IBM SPSS Statistics for Windows, Version 20.0 (IBM Corp., Armonk, NY, USA). Data were anonymized prior to statistical evaluation.

Descriptive statistics were used to summarize demographic and clinical characteristics. Categorical variables were expressed as frequencies and percentages. Differences in vaccination status (fully vaccinated, partially vaccinated, and not vaccinated) across clinical and sociodemographic variables were assessed using the Pearson chi-square (χ^2^) test. When expected cell counts were small, Fisher’s exact test or Monte Carlo simulation was applied as appropriate. Effect sizes for significant associations were estimated using Cramer’s V. A two-sided *p*-value < 0.05 was considered statistically significant.

For analytical purposes, parental age was categorized into four groups (20–29, 30–39, 40–49, and ≥50 years). Parental education levels were classified as low (no formal education, primary or middle school), medium (high school), and high (university or higher).

For regression analyses, the primary outcome variable was defined as incomplete or no National Immunization Program (NIP) vaccination (partially vaccinated and not vaccinated combined) versus full vaccination.

Univariable binary logistic regression analyses were first performed to examine associations between incomplete or no NIP vaccination and maternal age, paternal age, maternal education level, paternal education level, and number of children in the household. Odds ratios (ORs) with 95% confidence intervals (CIs) were calculated using appropriate reference categories (the youngest age group for parental age and the highest education level for parental education).

A multivariable binary logistic regression model was subsequently constructed to evaluate independent associations with incomplete or no NIP vaccination. The model included maternal age, paternal age, parental education levels, and the number of children in the household. These variables were selected based on clinical relevance and prior evidence suggesting potential associations with vaccination behaviors. During development of the multivariable logistic regression model, all parental age categories were initially evaluated. However, inclusion of the ≥50-year category yielded unstable parameter estimates in the preliminary multivariable analyses. Therefore, this category was not retained in the final adjusted model to improve overall model stability and interpretability. Adjusted odds ratios (aORs) with 95% CIs were reported.

Associations between motor development level (normal motor development, mild–moderate motor impairment [GMFCS I–III] and severe motor impairment [GMFCS IV–V]) and vaccination status were initially assessed using chi-square analysis. A separate logistic regression model including GMFCS categories was constructed to estimate the association between motor impairment severity and incomplete or no vaccination, using normal motor development as the reference group. No additional covariates were included in this model; therefore, the resulting odds ratios represent unadjusted estimates.

Parental age and education were additionally evaluated for their association with uptake of at least one non-NIP (optional) vaccine using chi-square analysis. For selected binary comparisons (e.g., Down syndrome versus all other diagnostic groups for influenza vaccine uptake), odds ratios with 95% confidence intervals were calculated.

Because complete vaccine refusal was infrequent, separate multivariable modeling using refusal as an isolated outcome was not performed.

### 2.6. Ethical Approval

This study was conducted in accordance with the principles of the Declaration of Helsinki and was approved by the Turkish Ministry of Health and the local Ethics Committee of Zeynep Kamil Maternity and Children’s Training and Research Hospital (Istanbul, Türkiye) (approval date: 27 August 2025; approval number: 101). Written informed consent was obtained from parents or legal guardians prior to participation, as data were collected using a brief questionnaire administered during routine clinical visits.

## 3. Results

### 3.1. Demographic and Clinical Characteristics of the Cohort

A total of 545 pediatric patients were included in the study, of whom 339 (62.2%) were male and 206 (37.8%) were female. The study population consisted of children with a broad spectrum of chronic neurological and neurodevelopmental disorders. The most frequent diagnosis was autism spectrum disorder, accounting for 39.3% of the cohort, followed by cerebral palsy (24.2%), Down syndrome (17.4%), and neurogenetic disorders (11.2%). Less common diagnoses included spina bifida (with/without hydrocephalus), neurometabolic disorders, neuromuscular disorders, and isolated hydrocephalus. Detailed demographic characteristics of the study population are summarized in Table 1.

Among the 132 children with cerebral palsy, 54 (40.9%) had comorbid epilepsy, while 78 (59.1%) did not. Of the 16 children with spina bifida, 15 (93.8%) had associated hydrocephalus.

### 3.2. Immunization Status According to Diagnostic Group

Vaccination status by diagnostic group is summarized in Table 2.

Not vaccinated indicates a complete refusal of routine childhood immunizations.

Percentages for a and b are calculated within the partially vaccinated group for each diagnostic category.

Immunization status (fully vaccinated, partially vaccinated, and not vaccinated) was significantly associated with diagnostic group (χ^2^ = 67.95, df = 14, Monte Carlo *p* < 0.001), with a moderate effect size (Cramer’s V = 0.25). Full vaccination rates varied across diagnostic categories, with lower completion observed in autism spectrum disorder and neuromuscular disorders, and higher completion in Down syndrome and other neurogenetic disorders. Partial vaccination was more frequently recorded among children with autism spectrum disorder, cerebral palsy, and spina bifida. Among partially vaccinated children, parental concern about potential neurological deterioration was the predominant reason for incomplete vaccination (94.1%), whereas physician-advised postponement accounted for 5.9% of cases. This pattern was observed across diagnostic groups, with parental concern regarding potential neurological deterioration remaining the predominant reason for partial vaccination, whereas physician-advised postponement was observed only in a limited number of cases (Table 2). Complete non-vaccination was uncommon overall but was present across several diagnostic categories; findings for isolated hydrocephalus should be interpreted with caution due to the small sample size (*n* = 3).

In a planned logistic regression analysis modeling full vaccination (fully vaccinated vs. not fully vaccinated) using Down syndrome as the reference group, the odds of full vaccination were significantly lower in autism spectrum disorder (OR 0.33, 95% CI 0.17–0.62, *p* < 0.001) and cerebral palsy (OR 0.41, 95% CI 0.21–0.81, *p* = 0.011), whereas no significant difference was observed for other neurogenetic disorders (OR 0.64, 95% CI 0.28–1.47, *p* = 0.29).

### 3.3. Uptake of Non-NIP Vaccines

Uptake of non-NIP vaccines across diagnostic groups is summarized in Table 3.

Receipt of at least one non-NIP vaccine was not significantly associated with diagnostic group (χ^2^ test, *p* = 0.089). When individual vaccines were examined, uptake of rotavirus and meningococcal vaccines was not significantly associated with diagnosis, whereas influenza vaccination showed a significant association with diagnostic group (χ^2^ test, *p* = 0.003). Overall uptake of non-NIP vaccines was low in the cohort. In a planned binary comparison (Down syndrome vs. all other diagnoses), influenza vaccination was significantly more frequent in Down syndrome (8/95 vs. 4/450; OR 10.25, 95% CI 3.02–34.80; *p* < 0.001).

### 3.4. Sociodemographic Factors Associated with Incomplete Vaccination

Analysis of parental characteristics demonstrated significant associations between maternal age (χ^2^ = 18.53, df = 6, *p* = 0.005) and paternal age (χ^2^ = 14.69, df = 6, *p* = 0.023) and NIP immunization status. Parental age was not associated with uptake of any non-NIP vaccine. In contrast, both maternal and paternal education levels were significantly associated with NIP immunization status (maternal education: χ^2^ = 17.97, df = 4, *p* = 0.0013; paternal education: χ^2^ = 22.01, df = 4, *p* = 0.0002) as well as with non-NIP vaccine uptake (maternal education: χ^2^ = 74.72, df = 2, *p* < 0.001; paternal education: χ^2^ = 61.27, df = 2, *p* < 0.001).

In univariable binary logistic regression analyses, maternal age, paternal age, parental education level, and number of children in the household were associated with incomplete or no NIP vaccination (Table 4).

In multivariable logistic regression analysis including maternal age, paternal age, parental education levels, and number of children in the household, maternal age remained significantly associated with incomplete or no NIP vaccination, whereas paternal age, parental education levels, and number of children were not significantly associated after adjustment (Table 4).

### 3.5. Motor Impairment Severity and Vaccination Status

Of the 545 children included in the study, 251 (46.1%) had normal motor development, 163 (29.9%) had mild–moderate motor impairment, and 131 (24.0%) had severe motor impairment. In univariable analysis, a statistically significant difference in vaccination status was observed across motor development levels (Pearson χ^2^ = 13.28, *p* = 0.001), with incomplete or no vaccination more frequently noted among children with severe motor impairment (GMFCS IV–V). In a separate logistic regression analysis, mild to moderate motor impairment (GMFCS I–III) was associated with lower odds of incomplete or no vaccination compared with normal motor development (OR 0.44, 95% CI 0.27–0.71, *p* = 0.001). By contrast, severe motor impairment (GMFCS IV–V) did not show an independent association (OR 1.03, 95% CI 0.66–1.60, *p* = 0.89).

### 3.6. Timing of Vaccination Interruption Among Partially Vaccinated Children

Timing of vaccination interruption among partially vaccinated children according to diagnostic group is summarized in Table 5.

Among partially vaccinated children, the age period after which routine childhood immunization was no longer received differed significantly across diagnostic groups (χ^2^ = 47.45, df = 18, *p* < 0.001), with interruption after 48 months emerging as the most common pattern.

## 4. Discussion

### 4.1. Overall Immunization Status in a Clinically Vulnerable Population

In this study, conducted in a large, heterogeneous cohort of children with chronic neurological and neurodevelopmental disorders, full adherence to the National Immunization Program was observed among 72.1% of participants. A further 25.0% were partially vaccinated, and 2.9% had never received routine childhood vaccines. These findings indicate that, despite high national immunization coverage reported at the population level, continuity of routine vaccination may be interrupted in clinically vulnerable pediatric subgroups [1,2,10]. Comparable disparities in immunization coverage and timeliness have been documented in cohorts of children with chronic diseases, in which both the coverage and timing of routine vaccines exhibited heterogeneous patterns across medically complex groups [11].

Previous studies from different countries have generally reported high acceptance of routine childhood vaccination among children with chronic neurological conditions; however, these studies predominantly focused on selected diagnostic groups or assessed immunization status using dose-based or age-appropriate definitions [6,7,9]. In contrast, defining incomplete immunization as discontinuation of the routine vaccination schedule may provide additional insight into real-world parental decision-making and clinical follow-up practices in tertiary pediatric neurology settings. From this perspective, our findings suggest that national immunization coverage indicators may not fully reflect vaccination patterns observed in specialized clinical populations. The variability observed across diagnostic categories further supports the need to interpret immunization status within diagnosis-specific clinical contexts rather than as a uniform phenomenon within the CNDD population.

### 4.2. Diagnosis-Specific Patterns of Incomplete Immunization

In this cohort, immunization completeness varied across diagnostic groups, suggesting that incomplete vaccination is shaped by diagnosis-specific clinical and developmental contexts rather than representing a uniform pattern among children with chronic neurological and neurodevelopmental disorders. Across most diagnostic categories, partial vaccination was more frequent than complete vaccine refusal, indicating that interruptions in routine immunization may more often reflect postponement or discontinuation decisions than outright rejection [5,12,13].

Children with autism spectrum disorder (ASD) showed one of the highest proportions of partial vaccination in the present cohort. This pattern suggests that vaccination decisions in this group may intersect temporally with the diagnostic process rather than reflecting early or generalized vaccine refusal. Population-based studies have demonstrated reduced vaccination uptake not only among children diagnosed with ASD but also among their younger siblings, indicating that vaccine-related concerns may extend to family-level decision-making following diagnosis [8]. In this context, heightened parental vigilance and perceptions of neurodevelopmental vulnerability after diagnosis may contribute to more cautious continuation of routine immunization, particularly with respect to later booster doses, even in the absence of established medical contraindications or causal evidence linking vaccines to ASD [14]. Multivariable analysis was consistent with descriptive findings, with children with ASD showing lower odds of full vaccination compared with those with Down syndrome.

Children with cerebral palsy (CP) demonstrated intermediate rates of complete vaccination, with partial immunization remaining common in this group. Previous studies have reported delayed or incomplete vaccination in children with CP despite the absence of disease-specific contraindications, suggesting that clinical heterogeneity, comorbidities, and competing medical priorities may influence vaccination continuity [6,9]. In our cohort, motor impairment severity showed a differentiated pattern: although descriptive analyses suggested variation across functional levels, regression modeling indicated that children with mild–moderate motor impairment had lower odds of incomplete or no vaccination compared with those with normal motor development, whereas severe motor impairment did not retain an independent association after adjustment. Taken together, these findings suggest that motor impairment severity alone may not fully explain vaccination interruption in children with cerebral palsy. Previous studies have proposed that broader clinical complexity and healthcare-related factors may also play a role in vaccination continuity in this population [6,9].

By contrast, children with Down syndrome in our cohort exhibited relatively high rates of complete vaccination, although partial immunization was observed in a subset. Earlier reports have noted lower vaccination rates and increased parental hesitancy in this group despite clear immunization recommendations, highlighting the potential role of frequent medical visits, intercurrent illnesses, and practical challenges in maintaining vaccination schedules [15,16]. In our cohort, influenza vaccination uptake was higher among children with Down syndrome compared with other diagnostic groups. This may reflect the structured medical follow-up and increased healthcare contact typically observed in this population. This difference may partly explain why our findings contrast with some earlier reports describing lower vaccination coverage in children with Down syndrome. The tertiary pediatric neurology setting of the present study and the regular multidisciplinary follow-up commonly provided to these children may facilitate greater adherence to recommended immunization schedules. In addition, children with Down syndrome have an increased susceptibility to respiratory tract infections and may exhibit immune system abnormalities that contribute to higher infectious morbidity [16,17]. Greater awareness of these risks among healthcare providers and caregivers may also contribute to the higher influenza vaccine uptake observed in this subgroup.

Overall, these findings suggest that incomplete immunization among children with chronic neurological and neurodevelopmental disorders follows diagnosis-specific trajectories shaped by clinical context and the timing of diagnostic and therapeutic processes. Recognition of this heterogeneity may support diagnosis-sensitive counseling and continuity of routine immunization during ongoing neurological follow-up [5,12].

### 4.3. Timing and Context of Incomplete Vaccination

The diagnosis-specific differences observed in the age period after which routine childhood immunization was no longer received suggest that interruptions in vaccination among children with neurological and neurodevelopmental disorders are shaped not only by disease burden but also by the developmental stage and clinical context in which diagnostic concerns arise. Previous studies indicate that vaccination behaviors in medically vulnerable pediatric populations are influenced by clinical complexity, parental risk perception, and healthcare encounters, rather than uniform vaccine refusal patterns [5,12,13]. Accordingly, incomplete vaccination in this population appears to follow distinct temporal patterns across diagnostic groups, as supported by the significant between-group differences observed in our analysis.

In the present cohort, interruption of routine childhood immunization from 48 months onward was more frequently observed among children with autism spectrum disorder (ASD). This pattern indicates that vaccination may be maintained during infancy and early childhood but becomes incomplete during the preschool booster period, when vaccines scheduled at and after 48 months would typically be administered. The observed pattern suggests that vaccination discontinuation may occur during the preschool booster period; however, the reasons underlying this finding cannot be determined from the present data, as neither parental perceptions nor the timing of ASD diagnosis were evaluated. Population-based studies have shown reduced vaccination uptake not only among children with ASD but also among their younger siblings, indicating that diagnostic experiences may shape family-level immunization decisions [8]. In addition, qualitative and survey-based studies report higher levels of vaccine-related concern among parents of children with ASD, often driven by perceived temporal associations or concerns about symptom exacerbation rather than evidence-based contraindications [14,18]. However, given the cross-sectional design of this study, the observed association should be interpreted as descriptive rather than causal, and the current data do not allow definitive conclusions regarding the temporal relationship between ASD diagnosis and subsequent vaccination interruption.

By contrast, earlier interruption of routine childhood immunization, reflected by lack of vaccination from earlier age periods, was more commonly observed in conditions such as spina bifida. This pattern may be more closely associated with medical complexity in early life, including surgical interventions, recurrent hospitalizations, and intensive clinical follow-up during infancy. Previous studies in children with chronic neurological disorders have shown that frequent hospitalizations and intercurrent illnesses may contribute to delays or interruptions in routine vaccination schedules, often in the context of physician-recommended caution rather than parental vaccine refusal [3,13]. Nevertheless, these interpretations should be considered hypothesis-generating, as the present design does not allow causal inference regarding the drivers of early vaccination interruption.

Although vaccine-specific attitudes and MMR vaccine uptake were not evaluated in the present study, extensive epidemiological evidence has consistently demonstrated no causal association between routine childhood vaccination, including measles–mumps–rubella (MMR), and autism spectrum disorder. Large-scale population studies and meta-analyses have confirmed the absence of such a relationship, and these findings have been reaffirmed by international health authorities [19,20]. This evidence provides important context for understanding vaccine-related concerns that have been reported in the ASD literature.

Taken together, these findings suggest that interruptions in routine childhood immunization among children with neurological and neurodevelopmental disorders are shaped by diagnosis-specific timing and clinical trajectories, rather than reflecting uniform vaccine refusal. Recognition of these temporal patterns may support diagnosis-sensitive counseling and help maintain continuity of routine immunization during critical periods of clinical evaluation and care [5,12].

### 4.4. Parental and Household Characteristics Associated with Incomplete Vaccination

In this cohort of children with chronic neurological and neurodevelopmental disorders (CNDD) followed in a tertiary pediatric neurology setting, routine immunization completeness varied according to several parental and household characteristics, including parental age, parental education level, and household size. Similar sociodemographic patterns have been reported across different pediatric populations, particularly among medically vulnerable children, and are generally interpreted as contextual influences on vaccination continuity rather than direct indicators of vaccine refusal [12,13,21]. However, after adjustment for other parental and household variables, only maternal age remained significantly associated with incomplete or no NIP vaccination.

Lower parental education levels were associated with higher proportions of incomplete vaccination in the present cohort. Previous population-based studies and systematic reviews have reported similar findings, suggesting that lower educational attainment may influence vaccination continuity through reduced health literacy, limited access to reliable health information, and greater difficulty navigating complex healthcare recommendations [12,13,21,22]. However, this relationship was attenuated after multivariable adjustment and was no longer statistically significant.

Maternal age remained significantly associated with vaccination completeness, with older maternal age groups demonstrating lower odds of incomplete or no vaccination. Previous studies have suggested that parental age may influence adherence to routine immunization and other preventive healthcare practices through differences in healthcare experience, access to health information, and engagement with healthcare systems [21,22].

Household size was also associated with vaccination status, with larger families exhibiting higher rates of incomplete immunization. Earlier studies have suggested that increased caregiving demands and logistical barriers in larger households may complicate adherence to routine vaccination schedules, particularly among families caring for children with chronic conditions [12,13]. However, this association was attenuated after multivariable adjustment and was no longer statistically significant.

Taken together, these findings suggest that selected parental and household characteristics may contribute to variability in the continuity of routine immunization among children with CNDDs. Recognition of these factors may support individualized counseling and follow-up strategies during ongoing neurological care [12,13,21].

### 4.5. Clinical Interpretation of Partial Versus Complete Non-Vaccination

In the present cohort, partial vaccination was substantially more common than complete non-vaccination among children with chronic neurological and neurodevelopmental disorders (CNDD) [6,7,9,11]. Among children with chronic or medically complex conditions, interruptions to routine immunization more commonly occur as delayed or discontinued vaccination at later stages of the schedule rather than as early or complete vaccine refusal [11,23].

Studies in pediatric populations with chronic disease have shown that partial or delayed vaccination is more common than complete non-vaccination, reflecting the effects of clinical complexity, frequent healthcare encounters, and competing medical priorities on vaccination continuity [6,7,24]. Vaccine-related behaviors have also been described as existing along a continuum ranging from timely acceptance to delayed or selective vaccination, rather than as a simple accept–refuse dichotomy [5,12,13,25].

In the present cohort, the predominance of partial vaccination across multiple diagnostic categories may reflect shared contextual and clinical factors rather than diagnosis-specific contraindications. Comparable observations have also been reported in children with special healthcare needs and chronic neurological conditions, where partial vaccination has been viewed as an area that may benefit from continued clinical engagement and follow-up [24,26].

Partial vaccination may represent a clinically distinct and potentially addressable pattern, as families who remain engaged with healthcare services may be more receptive to counseling and follow-up regarding delayed or outstanding vaccine doses [12,13]. Evidence from population-based surveys and clinical studies suggests that continued provider engagement and diagnosis-sensitive communication may support completion of delayed vaccinations, particularly in children with chronic health needs [23,25]. In the present cohort, parental concerns about potential neurological deterioration accounted for the vast majority of partial vaccination cases (94.1%), whereas physician-advised postponement accounted for only a small proportion (5.9%). Although physician-related delays were relatively uncommon, this finding underscores the importance of ensuring that healthcare professionals remain informed about current evidence-based vaccination recommendations for children with chronic neurological and neurodevelopmental disorders. Continued educational initiatives, together with diagnosis-specific vaccination counseling and effective communication between pediatric subspecialists and primary care providers, may help prevent unnecessary postponement of vaccinations and support continuity of routine immunization. Overall, these findings suggest that partial vaccination should be considered separately from complete vaccine refusal in children with CNDDs [5,12]. Recognizing this distinction may help support individualized strategies aimed at maintaining vaccination continuity during long-term neurological follow-up [12,23].

### 4.6. Strengths and Limitations

Several limitations should be considered when interpreting these findings. Although multivariable logistic regression was performed for the combined outcome of incomplete or no NIP vaccination, the number of children with complete vaccine refusal was limited. Therefore, complete refusal could not be analyzed as a separate outcome, and reliable estimates for refusal-specific models could not be obtained. In addition, residual confounding cannot be excluded given the observational cross-sectional design. Although diagnostic category and motor impairment severity were evaluated in separate analyses, these variables were not included in the primary multivariable model. Given the relatively small numbers within several diagnostic subgroups and the limited number of outcome events in some strata, inclusion of all clinical and sociodemographic variables in a single multivariable logistic regression model may have resulted in unstable parameter estimates. Therefore, a more parsimonious multivariable model was considered an appropriate analytical approach for the primary analysis. Consequently, some adjusted odds ratio estimates were accompanied by wide confidence intervals and should therefore be interpreted with caution. Accordingly, residual confounding related to clinical characteristics cannot be completely excluded. Furthermore, some diagnostic subgroups, particularly isolated hydrocephalus, neuromuscular disorders, and neurometabolic disorders, contained relatively small numbers of participants. Therefore, subgroup-specific findings should be interpreted with caution and considered exploratory.

Vaccination status was assessed using routinely collected clinical records and parental reports. Although this reflects standard documentation practices in tertiary pediatric neurology settings, it may be subject to incomplete or variable reporting. In addition, the cross-sectional design precludes temporal inference, and observed associations should therefore be interpreted as descriptive rather than causal. Furthermore, potentially relevant factors such as age at diagnosis, timing of developmental regression, initiation of rehabilitation services, frequency of healthcare encounters, major medical interventions, broader parental attitudes, beliefs, and parenting characteristics were not systematically collected. These factors may have influenced parental vaccination decisions and, therefore, constitute potential sources of residual confounding. Although the overall participation rate was high, the diagnostic distribution of families who declined participation and children whose vaccination status could not be verified was not systematically evaluated. Therefore, differential non-participation across diagnostic groups cannot be completely excluded and may have influenced the observed diagnostic patterns. Future studies incorporating these psychosocial and behavioral determinants may provide a more comprehensive understanding of vaccine hesitancy and vaccination continuity in children with chronic neurological and neurodevelopmental disorders.

A major strength of this study is the evaluation of routine immunization status in a large and heterogeneous cohort of children with CNDDs followed at a single tertiary pediatric neurology center. The study population was derived from a tertiary center in Istanbul, a large metropolitan area with diverse sociodemographic characteristics, providing relevant insight into vaccination patterns among vulnerable children in urban settings. Although the study was conducted at a single tertiary pediatric neurology center in Istanbul, the institution serves as a referral center for patients from different regions of Türkiye. Nevertheless, multicenter studies including populations from different geographic regions would be valuable to further assess the generalizability of these findings.

The inclusion of a heterogeneous CNDD population allowed evaluation of both diagnosis-specific and shared patterns of incomplete vaccination within the same cohort, rather than limiting interpretation to a single neurological condition. Furthermore, defining incomplete vaccination as discontinuation of the routine immunization schedule, rather than dose-based coverage alone, provided clinically meaningful insight into real-world vaccination continuity during routine neurological follow-up.

## 5. Conclusions

This study shows that incomplete vaccination among children with chronic neurological and neurodevelopmental disorders (CNDD) is more commonly characterized by partial or delayed immunization than by complete vaccine refusal. Vaccination patterns varied across diagnostic groups and were associated with selected parental and household factors, highlighting the heterogeneity of immunization trajectories in this population. These findings suggest that interruptions in routine immunization should be interpreted within broader developmental and healthcare contexts rather than as a uniform manifestation of vaccine hesitancy. Recognition of diagnosis-specific patterns of incomplete vaccination may help identify opportunities for targeted counseling and support continuity of routine childhood immunization.

## Figures and Tables

**Table 1:** Demographic characteristics of the study population.

Diagnosis Group	*n* (%)	Male	Female	Mean Age (Months) ± SD	Range (Min–Max)
Autism spectrum disorder	214 (39.3)	170	44	101.1 ± 41.0	30–201
Cerebral palsy	132 (24.2)	78	54	100.1 ± 50.6	13–206
Down syndrome	95 (17.4)	40	55	84.3 ± 49.2	13–209
Other neurogenetic disorders (excluding Down syndrome)	61 (11.2)	29	32	83.4 ± 43.2	18–203
Spina bifida ^a^	16 (2.9)	9	7	93.1 ± 56.5	21–198
Neurometabolic disorders	15 (2.8)	7	8	84.4 ± 51.7	17–203
Neuromuscular disorders	9 (1.7)	5	4	133.8 ± 63.6	31–201
Hydrocephalus (isolated)	3 (0.6)	1	2	70.7 ± 51.0	15–115
TOTAL	545 (100.0)	339	206	95.6 ± 47.1	13–209

^a^ Of the 16 children with spina bifida, 15 (93.8%) had associated hydrocephalus.

**Table 2:** Immunization status and reasons for partial vaccination by diagnostic group.

Diagnosis Group	*n*	Fully Vaccinated *n* (%)	Partially Vaccinated *n* (%)	Not Vaccinated (Vaccine Refusal) *n* (%)
Autism spectrum disorder	214	140 (65.4)	70 (32.7) a: 70 (100) b: 0 (0)	4 (1.9)
Cerebral palsy	132	93 (70.5)	36 (27.3) a: 33 (91.7) b: 3 (8.3)	3 (2.3)
Down syndrome	95	81 (85.3)	11 (11.6) a: 9 (81.8) b: 2 (18.2)	3 (3.2)
Other neurogenetic disorders (excluding Down syndrome)	61	48 (78.7)	11 (18) a: 10 (90.9) b: 1 (9.1)	2 (3.3)
Spina bifida	16	10 (62.5)	5 (31.2) a: 4 (80) b: 1 (20)	1 (6.2)
Neurometabolic disorders	15	14 (93.3)	1 (6.7) a: 0 (0) b: 1 (100)	0 (0)
Neuromuscular disorders	9	6 (66.7)	2 (22.2) a: 2 (100) b: 0 (0)	1 (11.1)
Hydrocephalus (isolated)	3	1 (33.3)	0	2 (66.7)
TOTAL	545	393 (72.1)	136 (25)	16 (2.9)

Note: a: Partial vaccination due to parental concern about potential neurological deterioration. b: Partial vaccination due to physician-advised postponement of vaccination.

**Table 3:** Distribution of Non-NIP Optional Vaccines Across Diagnostic Groups.

Diagnostic Group	Total *n*	Any Non-NIP Vaccine *n* (%)	Rotavirus Vaccine *n*	Meningococcal Vaccine *n*	Influenza Vaccine *n*
Autism spectrum disorder	214	31 (14.5)	29	16	2
Cerebral palsy	132	16 (12.1)	15	15	2
Down syndrome	95	26 (27.4)	19	14	8
Other neurogenetic disorders	61	13 (21.3)	10	10	0
Spina bifida	16	2 (12.5)	2	0	0
Neurometabolic disorders	15	2 (13.3)	2	1	0
Neuromuscular disorders	9	1 (11.1)	1	1	0
Hydrocephalus (isolated)	3	0 (0)	0	0	0
TOTAL	545	91 (16.7)	78	57	12

**Table 4:** Logistic regression analyses of factors associated with incomplete or no NIP vaccination.

Univariable Logistic Regression Analysis				
Variable	Category	OR	95% CI	*p* Value
Maternal age	20–29 (ref)	1.00	—	—
	30–39	0.15	0.04–0.54	0.005
	40–49	0.34	0.12–0.93	0.035
	≥50	0.51	0.18–1.46	0.194
Paternal age	20–29 (ref)	1.00	—	—
	30–39	0.32	0.16–0.61	0.001
	40–49	0.44	0.22–0.86	0.016
	≥50	0.15	0.02–1.11	0.090
Maternal education	High (ref)	1.00	—	—
	Intermediate	2.27	0.90–5.73	0.082
	Low	4.26	1.77–10.27	0.001
Paternal education	High (ref)	1.00	—	—
	Intermediate	1.27	0.64–2.54	0.492
	Low	2.92	1.57–5.45	0.001
Number of children in household	≥3 (ref)	1.00	—	—
	1 child	0.51	0.30–0.87	0.017
	2 children	0.63	0.42–0.94	0.027
**Multivariable Logistic Regression Analysis**				
**Variable**	**Category**	**Adjusted OR (95% CI)**		***p* Value**
Maternal age	20–29 (ref)	1.00		—
	30–39	0.25 (0.08–0.77)		0.016
	40–49	0.62 (0.37–1.03)		0.064
Paternal age	20–29 (ref)	1.00		—
	30–39	1.04 (0.10–11.28)		0.973
	40–49	1.07 (0.62–1.83)		0.819
Maternal education	High (ref)	1.00		—
	Low	2.93 (0.93–9.24)		0.066
	Medium	2.35 (0.81–6.76)		0.114
Paternal education	High (ref)	1.00		—
	Low	1.67 (0.71–3.97)		0.243
	Medium	0.92 (0.41–2.05)		0.833
Number of children in household	≥3 (ref)	1.00		—
	1 child	0.81 (0.44–1.47)		0.479
	2 children	0.79 (0.52–1.22)		0.296

Abbreviations: OR, odds ratio; CI, confidence interval; ref, reference category. Note: Univariable binary logistic regression analyses were performed. The dependent variable was defined as incomplete or no NIP immunization (partial or no vaccination) versus full vaccination. Odds ratios are presented relative to the reference category for each variable.

**Table 5:** Age period after which routine childhood immunization was no longer received among partially vaccinated children, by diagnostic group.

Diagnostic Group	From 12 Months Onward	From 24 Months Onward	From 48 Months Onward	Interrupted After 48 Months
Autism spectrum disorder	0	16	7	47
Cerebral palsy	0	13	9	14
Down syndrome	0	3	2	6
Other neurogenetic disorders (excluding Down syndrome)	0	5	1	5
Spina bifida	1	3	1	0
Neurometabolic disorders	0	1	0	0
Neuromuscular disorders	0	2	0	0
Total	1	43	20	72

Footnote: Values represent the number of children. Categories indicate the age period after which routine childhood immunization was no longer received: children in the “From 12 months onward,” “From 24 months onward,” and “From 48 months onward” categories did not receive routine vaccines scheduled from these respective ages onward. “Interrupted after 48 months” indicates that vaccination continued beyond 48 months but was not subsequently completed. Only partially vaccinated children were included.

## Data Availability

The data presented in this study are available from the corresponding author on reasonable request. The data are not publicly available due to patient privacy and ethical restrictions.

## Supplementary Materials

The following supporting information can be downloaded at: https://www.mdpi.com/article/10.3390/children13070891/s1, Table S1: National Immunization Schedule of Türkiye used in this study (adapted from the Republic of Türkiye Ministry of Health).

## Abbreviations

CNDD	Chronic neurological and neurodevelopmental disorders
GMFCS	Gross Motor Function Classification System
NIP	National Immunization Program
