# Supplementary material for: Immunization Patterns of Children with Chronic Neurological and Neurodevelopmental Disorders: A Cross-Sectional Study from Istanbul
Source: Children (Basel). 2026 Jul 2;13(7):891. doi: 10.3390/children13070891 (PMC13406390; doi:10.3390/children13070891)
Supplement: Supplementary file 1 [file children-13-00891-s001.zip › children-4360290-supplementary.pdf]

Table S1. Turkish National Immunization Program Schedule Used in This Study

**Vaccines**	**At birth**	**1 month**	**2 months**	**4 months**	**6 months**	**9 months**	**12 months**	**18 months**	**24 months**	**48 months**	**13 years**
**Hepatitis B**	I	II			III						
**BCG**			I								
**DTaP-Hib-IPV**			I	II	III			R			
**PCV13**			I	II			R				
**OPV**					I			II			
**MMR**							I			II	
**Varicella**							I				
**Hepatitis A**								I	II		
**DTaP-IPV**										R	
**Td**											R

BCG, Bacillus Calmette–Guérin; DTaP-Hib-IPV, diphtheria, tetanus, acellular pertussis, Haemophilus influenzae type b, inactivated poliovirus; PCV13, 13-valent pneumococcal conjugate vaccine; MMR, measles–mumps–rubella; OPV, oral poliovirus vaccine; Td, tetanus–diphtheria. I, first dose; II, second dose; III, third dose; R, booster dose.

Adapted from the Republic of Türkiye Ministry of Health National Immunization Program.
